# Psychiatric Aspects of Organ Transplantation

**Published:** 2011-02-01

**Authors:** G. Kalra, A. Desousa

**Affiliations:** 1 *Assistant Professor, *; 2 *Consultant Psychiatrist, Visiting Honorary Consultant, Department of Psychiatry, Lokmanya Tilak Municpal Medical College and General Hospital Mumbai, India*

**Keywords:** Organ transplantation, Donor, Recipient, Psychiatric aspects, Organs

## Abstract

Surgical transplantation of human organs from deceased as well as living donors to sick and dying patients began after the Second World War. Over the past 50 years the transplantation of human organs, tissues and cells has become a worldwide practice which has extended, and greatly enhanced the quality of hundreds of thousands of lives. The field of transplantation medicine provides an important chance for liaison between psychiatric professionals and other transplant physicians and surgeons. The discrepancy between the ever-increasing demand for organs but the decreasing supply makes it important to evaluate and prioritize individuals who are in dire need of the organ. However, this also gives rise to certain ethical questions. The following paper discusses various psychiatric aspects of organ transplantation in general.

## INTRODUCTION

Organ transplantation has by far been a very important milestone in the field of medicine. This is probably the only procedure that offers more hope of life for people suffering from a wide range of diseases that lead to end-stage organ failure and death than any other medico-surgical procedure. There is a huge demand for human organs for transplantation. However, one has to understand that the demand far exceeds the supply of the organs. A great deal of progress has been happening in this field from the time Thomas E. Starzl, the father of transplantation, performed the first human liver transplant in 1963 and the first successful liver transplant in 1967.

Psychiatric comorbidity in patients undergoing organ transplantation is an important issue and results in significant morbidity and mortality presenting a unique opportunity for psychiatric involvement in the care of medically ill patients. Transplantation medicine has expanded over the past decade and consultation-liaison psychiatrists play an important role in helping patients and their families deal with the plethora of psychosocial issues that may be involved in the procedure. This article discusses the importance of the role of psychiatrist in the transplant team. 

**ANCIENT TRANSPLANTATION**

The idea of people sharing body parts among themselves or with animals is as old as mankind. Various mythologies have remarkably similar tales of magical replacement of different body parts, usually with supernatural interventions. The cause and cure of disease and illness and the outcomes of various injuries were believed to be in the hands of gods and deities, and hence it was also assumed that supernatural forces could lift illnesses, ensure recovery from injury, and also replace lost tissue or organ [1]. If one looks at the great Hindu epics like *Ramayana* and *Mahabharatha*, one can easily see instances of organ transplantation, *e.g.*, Lord *Ganesha* had the head of a baby elephant.

One also comes across similar characters with the physical attributes of other creatures in Greek mythology. Some were animals, such as *Pegasus—*the winged horse—or the *Chimaera*, which had a lion’s head, a goat’s body and a serpent for a tail. The three *Gorgons* were fearsome female monsters with wings and writhing snakes for hair. The *Pan* was a Satyr with horns, a goat’s tail and hooves for feet [2]. The mermaids have a broad representation in folklore and literature around the world, and again represent a type of xenotransplant (a human-animal hybrid), with the head and torso of a human female and the remaining body of a fish. Another mythological creature depicted as a recumbent feline with a human head is the Greek *Sphinx*. In the Christian tradition, such miraculous transplantations were carried out by Christ and his saints. One instance described Christ replacing a slave’s ear that was cut off during Christ’s arrest, and Saint Peter also made such tissue restorations [3]. The best known legends of all are the miracles involving the twins Saint Cosmas and Saint Damian, who replaced a gangrenous leg [4]. Some reports of surgical reconstruction of the nose by grafting skin flaps date back to somewhere between 800 and 400 BC in ancient India, as reported by the Indian surgeon *Susruta* in his treatise *Sushruta Samhita*. This operation developed because of war injuries and the common punishment of cutting off noses for any wrongdoings that may have been done [2].

**TYPES OF ORGAN TRANSPLANTATION**

Donors can be classified differently, for instance, genetically-related donors, emotionally-related donors, Good Samaritan donors, vendors, and organ exchangers [5]. Different organs have been transplanted from the time of Starzl. Kidney continues to be the organ most frequently transplanted [6]. Out of all the organ transplantations, heart transplant seems to be the one that has and can get people deeply involved. The reasons for the same may include the radical nature of the procedure, and also the fact that heart is seen by some solely as a physiological pump, but by others as the symbolic seat of love and loyalty [7-8]. The psychiatric implications of closed- and open-heart surgery have been extensively documented. Of particular relevance was Kimball’s identification of patterns of emotional reaction in patients who were assessed pre-operatively, which were found to have predictive value. Of the four groups that he identified, namely, a) the adjusted, b) the symbiotic, c) the depressed, and d) those denying anxiety, it was the members of the latter two groups who caused further concern for the mental-health professionals. Depressed patients had a high post-operative mortality rate, while those who denied anxiety had a high incidence of post-operative psychiatric complications [9].

**DEMAND** ***VS*** **SUPPLY**

The demand for organs for transplantation is ever increasing. However, there is also simultaneously a shortage of organs. A common reason, perhaps for this scenario, may be the poor motivation in people to donate organs. Individuals may fear losing their own body parts or rather the integrity of their body and soul after death, for which they may refrain from allowing organ donation. On the contrary, there are people who donate organs with the notion that their body parts continue to “live” even after they die! Treating physicians may not provide proper information to people about the possibility and benefits of organ donation. They may ignore asking family members if they would consent to donating organs when their loved one dies.

An important controversy that comes off as an offshoot of this discrepancy between demand and supply is the responsibility to ensure that a scarce lifesaving resource is allocated appropriately to those in real need of it and not only to those who can afford to have it.

**PSYCHIATRY IN TRANSPLANTATION: THE INTERFACE**

**Figure 1:**
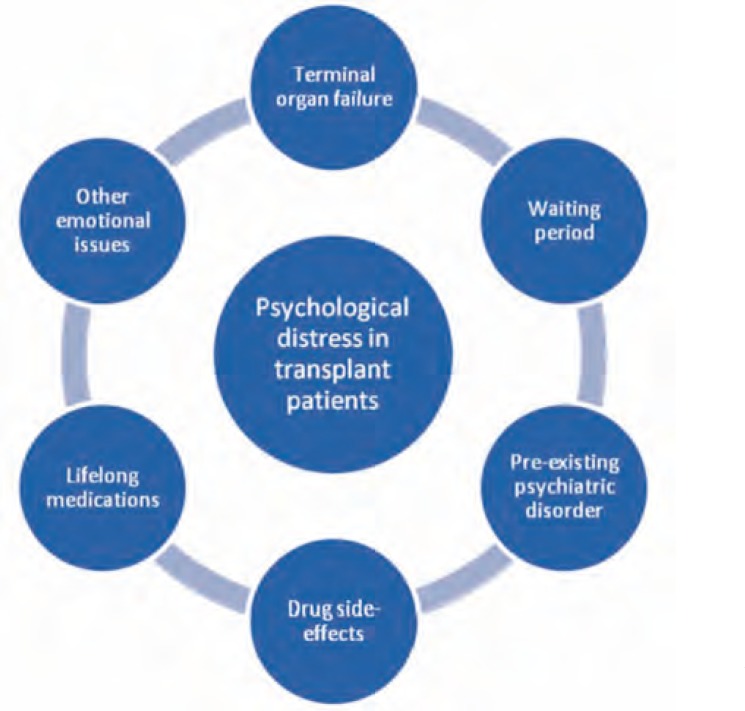
Factors in psychological distress in transplant patients

Recent years have witnessed a shift of the organs which can be transplanted leading to a shift of the challenges faced by the transplant team. The therapeutic procedures have also become more sophisticated requiring more specializations to get involved in the whole process. One may wonder what a psychiatrist’s role in the transplantation team may be. The very thought that an important part of one’s body has stopped working and is now needed to be replaced by someone else’s part may be difficult to accept and may act as a stressor for the patient, among other factors (Fig. 1). Psychological distress is common in patients undergoing transplantation with a range of emotions emerging in them (Table 1). A long waiting period for an appropriate donor may lead to distress [10] as much as complications resulting from various medical procedures and drug treatments [11]. The unpredictable outcome of the transplantation procedures creates a sort of fertile emotional soil for psychiatric complications [12]. On the other hand, the need for transplantation may also arise in previously psychologically ill patients. Therefore, the need of the mental health professional arises. Psychiatrists working with such individuals may have to deal with issues ranging from something as minor as anxiety about the surgical procedure to the fear of death and organ rejection.

**Table 1:** Psychological issues in pre-transplant patients

Distress from waiting for the appropriate matching donor
Loss of control over their lives due to rapidly declining lives [13]
Anxiety
Guilt
Irritability [13]
Anger
Denial
Hostility, Uncooperativeness, hypervigilance [13]
Feeling of uncertainty
Fear of death and dying

The psychiatrist has multiple roles on the transplant team, beginning with the transplantation psychiatry consultation (TPC). It addresses such issues as risks of exacerbation or recurrence of a psychiatric illness, pharmacokinetic and pharmacodynamic considerations due to organ failure, potential drug interactions involving psychotropic and immunosuppressant medications, adequacy of support system, history of medical compliance, emotional and cognitive preparedness for transplantation, mental status findings supplemented by standardized cognitive testing and psychosocial rating instruments, and decision-making capacity [14].

As a transplant team consultant, the psychiatrist treats peri-operative anxiety, depression, and organic brain dysfunction and addresses medical and ethical aspects of patient selection [15]. Allograft rejection and complications of immunosuppressant therapy are often associated with considerable stress, so availability of psychiatric consultation is a necessity.

**PRE-TRANSPLANT ASSESSMENT**

This part of the assessment can be challenging as the patients tend to present themselves in a socially acceptable manner to the transplantation team [16]. The main goal of pre-transplant psychiatric assessment is to identify any psychiatric morbidity that may interfere with the patient’s understanding of the transplant procedure, potential risk factors that may result in post-operative noncompliance and morbidity [17-19] and also to decide the plan of action for such identified at risk individuals [20-22]. In cases of organ transplantation, there has been an association between Axis I diagnoses and poorer psychosocial adjustment and health status, while Axis II diagnoses were associated with poorer compliance [23]. 

**Figure 2:**
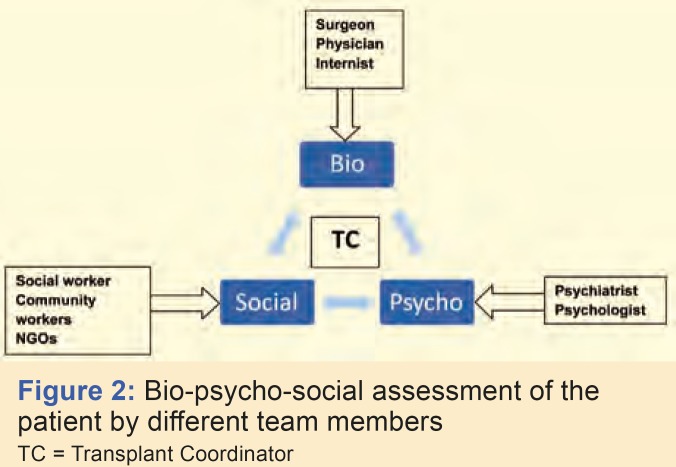
Bio-psycho-social assessment of the patient by different team membersTC = Transplant Coordinator

The screening and evaluation methods vary depending on centers and also according to the solid organ type. Cardiac transplantation programs have the most stringent psychosocial criteria, while renal programs, the most lenient; liver transplant programs usually take a moderate position in using psychosocial criteria [24]. Wherever feasible, a bio-psycho-social approach in the assessment is advisable (Fig. 2) by the different members of the transplant team. Such an approach gives an overall picture of the patient and helps the team to get to know the patient better, thus improving the patient care. Various screening instruments such as the Transplant Evaluation Rating Scale (TERS) [25], the Psychosocial Assessment of Candidates for Transplant (PACT) [26], and Structured Interview for Renal Transplantation (SIRT) [27] may be used to assist with the psychiatric evaluation.

The various aspects of psychiatric assessment (Table 2) should focus on any present and/or past psychiatric problems. Enquiry should also be made into the family history of psychiatric complaints and this should incorporate history of Axis I, Axis II disorders, history of self harm, *etc*. A detailed assessment of personality may aid in the process, especially if there is a co-morbid substance use disorder. 

**Table 2:** Pre-transplant assessment

Psychosocial Assessment
Past psychiatric history
Current psychiatric symptoms/illness
Psychotropic use
Substance use history
Social support
Cognitive evaluation
Understanding & knowledge

Apart from this, live donors should be informed of the probable risks, benefits and consequences of donation in a complete and understandable fashion; they should understand the fact that they too will be left with only one or incomplete organ after the donation, and may land up in a similar crisis later on in life. If not them, this crisis could occur with other relatives. They should be legally competent and capable of weighing the information; and they should be acting willingly, free of any undue influence or coercion, that is, it should be an informed decision. Failing to ensure that the person consenting to the donation should not have been paid, coerced (either overtly or covertly) or exploited breaches professional obligations [28]. 

Along with assessment, it is also important to ensure that both patients and their families have understood the transplant process fully [29]. The family interactions and relationships have to be understood to know the patient’s social support system, which will be the primary support for the patient to fall back on after surgery. The family members should be allowed to ventilate their feelings about the surgery and the patient’s ordeal, especially when they have to watch a loved one suffer with such a serious condition with no certainty of life. Some parents may carry guilt about passing a familial disease that may have resulted in the organ failure and hence the need for the transplant. 

In particular, the assessing psychiatrist should talk about the patient’s feelings about death and dying and facilitate ventilation regarding the same. Another area that needs exploration in this phase is the relationship which the recipient shares with the donor and the basic motive (both conscious and unconscious) that lies beneath the patient’s decision to donate to that particular recipient. It is also necessary to assess the donor’s understanding of the recipient’s illness, the urgency and the necessity of the current surgical transplant. Researchers have used certain case vignettes to point out that the decision to donate might be motivated by attempts to make reparation for wrongs committed in the past or to secure a commitment from the recipient [30]. It has also been suggested that donors should not be permitted to donate in clinically hopeless situations [31]. 

In general to sum-up, all psychiatric evaluations should rule out any acute psychopathology such as acute psychosis, acute mania or depression, active suicidal ideations, and active substance use, all of which may hinder with the eligibility of the candidate for transplantation procedure. Clinical evaluation may make do with identifying any psychiatric illness; however, one may use various projective tests like Rorschach to detect any underlying subtle psychopathology or other tests like Minnesota Multiphasic Personality Inventory (MMPI) to know the personality profile of the patient. Proper documentation of the clinical findings on mental state examination (MSE) including the test findings is important. A baseline mental state examination followed by serial MSEs may help in future comparisons and need to be documented properly. 

Patients may have high levels of anxiety related to the surgical procedure which may fade away after the surgery [32-33]. Anxiety levels may be seen in up to 80% of individuals pre-transplant [34]. 

**POST-TRANSPLANT FOLLOW-UP**

The vital role of the psychiatrist does not end with the surgical procedure but continues even after it, as adjustment after the surgery can be a stressful experience [35] with new psychiatric illness arising afresh or the previously-existing illness exacerbating post-procedure. Medication-induced mood disturbances, especially depression may be more commonly seen in the first year after transplantation [36]. Some illness-related psychiatric disorders may abate post-operatively [37]. 

Compliance with treatment, especially the immunosuppressants, is a major issue that arises post-transplant and is associated with a high level of morbidity and mortality [38]. However, one can predict compliance probability in the pre-transplant phase by the regularity with which the patient follows up with the treating team. For example, the degree of adherence to dialysis can be taken as an indication of possible compliance post-transplant [39]. Compliance issues may come up especially in patients with personality disorders. Other factors implicated in non-compliance post-transplant include young patients [40], cosmetic side-effects of immunosuppressants like acne, hirsutism, alopecia, *etc* [41], financial constraints, depression, and psychotic disorder. In the post-transplant phase, the field of psycho-neuro-immunology comes into play. It is worth noting that quality of life (QOL) may have a decisive impact on survival in these patients. QOL in transplanted patients is generally higher than in similarly ill patients who have not been transplanted, but is worse than in the healthy population. 

**OTHER ISSUES**

People who need organ transplant often have to wait a long time for an appropriate donor to come. This waiting period may increase the feeling of uncertainty in the patient and lead to mixed feelings of the possibility of a better life post-transplant. In post-transplant period, rejection of the graft may lead to pronounced emotional disturbances in both recipient and living donor [39]. If the living donor is a spouse or a parent, such graft failure may lead to guilt and depression. Critical care setups are other areas where organ transplantation gains importance as discussed by some authors [42]. 

Another issue that crosses one’s mind is that of selling of organs by the poor in need of money, which is commonplace in the developing countries. A sanction to live organ donation may have fuelled this trend as it also places societal pressure on the family members to donate to their loved ones. People have asked questions as to how far should one go to help save other lives, by endangering one’s own life through organ donation? This concept of living organ donation is virtually unique in that a healthy volunteer is exposed to the risk of surgery solely for the benefit of another individual. This along with efforts to increase the supply of organs has given birth to xenotransplantation. However, even this is not free of any controversies and has pulled in animal activism and fear of introducing some deadly new virus into the population, to the fore. 

Transplantation raises a number of bioethical issues, such as the definition of “death,” when and how consent should be given for an organ to be transplanted, payment for organ donation, and organ trafficking. WHO guidelines ban any sort of payment for tissues or organs which is said to take unfair advantage of the poorest and most vulnerable groups, leading to profiteering and human trafficking [31]. 

Another critical issue which we find very relevant to the Indian scenario and in our patients is the issue of transplant from an unknown donor *vs* the issue of transplant from a relative close to them. With the joint family system, we find many relatives with a high altruistic quotient who are willing to give up their body organ to save another family member and would prefer to do so rather than accept an unknown donor. The fact that a relative or close family member has donated the organ seems to increase the emotional attachment and reduce certain primitive anxieties associated with the transplantation process.

**MANAGEMENT ISSUES**

Role of the psychiatrist

The involvement of psychiatrist in organ transplantation can be at two levels. One and the most common, is after the organ failure has already occurred that is when the patient is pre-transplant. However, one should not forget their involvement at a stage when organ failure is yet to develop as in the case of alcohol dependence. Psychiatrists can make an extra-effort to psycho-educate the substance dependent individuals regarding the health hazards of continuing with the substance use and the consequences thereafter. The highest priority, however, should be given to initiatives aimed at the implementation of the primary-care approach, with strong components of prevention and health promotion in order to reduce the diseases that lead to the need for transplants in the first place.

With live donation, particularly by unrelated donors, psychosocial evaluation is needed to guard against coercion of the donor or the commercialism, in which the mental health professional has a major role to play as has already been discussed. It should be ensured that the evaluation is carried out by an appropriately qualified, independent party. By assessing the donor’s motivation and the donor’s and recipient’s expectations regarding outcomes, such evaluations may help identify—and avert—donations that are forced or are actually paid transactions.

The psychiatrist forms an important member of a transplant team ( Table 3 ), which should preferably be multidisciplinary [ 43 - 45 ]. It requires collaboration with multiple disciplines such as the operating surgeons, the chemotherapy unit, the psychologist, the social workers, the rehabilitation unit, *etc* .

**Table 3:** The multi-disciplinary transplant team

Transplant surgeon
Internists and sub-specialists
Psychiatrist and psychologist
Transplant coordinators/nurses
Social worker
Ethics committee me mbers

Psychiatrists can also take the lead in spreading awareness and educating people about the importance of organ donation and help in the set up of a community resource that is built on voluntary, unpaid donations of organs, tissues and cells and to which, all have equitable access. Group therapy sessions may be conducted or supervised by psychiatrists. Peer support groups with both donors and recipients may be beneficial. Family members of either the donor or recipients can form support groups for other patients and their families in need of psychological cushioning. Participating in such groups has been shown to increase compliance, and increase the sense of control [ 46 - 47 ]. Research suggests that education and psychosocial support during the transplant process improve the patient’s return to work and leisure activities [ 48 ].

The treating psychiatrist has to be careful about the drug interactions as the patient is likely to be on multiple drug regimen both pre- and post-transplant. The cytochrome P450 inhibiting action of the selective serotonin reuptake inhibitors (SSRIs) like fluvoxamine, and fluoxetine has to be kept in mind, as it could increase the plasma levels of immunosuppressants and hence lead to their toxicity. One has to be also aware of the interactions between *Hypericum perforatum* (St. John’s wort), a herbal extract used widely as a folk remedy for depression, and cyclosporine—the blood levels of the latter being decreased significantly by the former [ 49 ]. Immunosuppressant medications may lead to various side effects, some of which may mimic serious neuro-psychiatric conditions [ 50 ]; others include mood swings, sleep disorders, sexual dysfunction, and cognitive dysfunction [ 51 ]. The treating professional also has to take care of side-effects of the prescribed medications. Many of the newer psycho-pharmacologic agents result in weight gain and abnormalities of the regulation of glucose as side effects. These side effects need to be considered when treating pancreas and islet cell transplant recipients with psychiatric disorders [ 52 ].

Not all patients require psychopharmacological intervention; some of them may do well with psychotherapeutic approach. Religious and spiritual support can also help the patient in coping with the whole procedure. Authors have pointed that religious coping was associated with better adjustment in the short- and long-term for both the patient and their significant others [ 53 ]. An integrative approach combining aspects of various psychotherapeutic approaches throughout the transplant process always works best [ 54 ]. Electroconvulsive therapy (ECT) may also be used as a first line treatment to treat depression with suicidal ideations in some patients for quick response [ 55 ]. Finally, the psychiatrist may have to help the patient deal with the disappointment of organ rejection in case it occurs.

The role of celebrities as brand ambassadors in spreading the word about organ donation cannot be under-estimated and they can be used to sensitize the general public about organ donation.

**FUTURE CONSIDERATIONS**

The future of organ transplantation will depend on not only resolving the ethical and psychosocial issues, but also on increasing the supply of organs or finding more and more organ substitutes to keep up with the ever-increasing demand. One also has to take a relook at the absolute and relative contraindications for organ transplantation. Researchers have pointed out how the absolute contraindications may become less rigidly defined as more clinical experience is gained [ 56 - 57 ], as also the complete omission of the relative contraindications with progress in psychosocial research. But at the same time, they also warn the possibility of movement in either direction, in that what is relative now may become absolute with more experience. An example of a relative contraindication that may become more rigid is personality disorder. One also needs to wait and see what tissue engineering has to offer for organ transplantation and how it affects the prospects of live organ donors in future.

## CONCLUSION

Transplantation medicine is one of the most challenging and complex areas of modern medicine. Although the number of transplantations each year has grown rapidly over the past two decades, the demand for transplantation using human cells, tissues and organs has also increased significantly, resulting in a continuing shortage of human material, particularly organs. This has given way to newer avenues for exploration, which include xenotransplantation, tissue engineering, and even commercial transplantation in order to profit from the sale of organs removed from vulnerable citizens. Psychiatry’s involvement in the area of transplantation has come a long way from just being involved in assessing the patients pre-transplant to educating public about the importance of organ donation. Carefully designed and consistently implemented campaigns of advocacy that target all population groups, can help to increase public awareness that donation and transplantation are valuable and necessary.
